# A Review on the Effects of Aerobic Exercise on Immune Dysregulation in Polycystic Ovarian Syndrome

**DOI:** 10.7759/cureus.72537

**Published:** 2024-10-28

**Authors:** Shannon N Smith, Sierra Scott, Sydney Elness, Jonathan R Raymond-Lezman, Suzanne I Riskin

**Affiliations:** 1 Department of Foundational Sciences, Nova Southeastern University Dr. Kiran C. Patel College of Osteopathic Medicine, Clearwater, USA; 2 Department of Foundational Sciences, Nova Southeastern University Dr. Kiran C. Patel College of Osteopathic Medicine, Davie, USA

**Keywords:** exercise, immunological dysregulation, lifestyle modifications, over-inflammatory, pcos, pcos and exercise, women's health

## Abstract

Polycystic ovarian syndrome (PCOS) is the most common endocrine disorder of reproductive-aged females. However, much of its pathophysiology is not well investigated or understood. Research has demonstrated an association between PCOS and metabolic dysfunction, including insulin resistance. The aim of this review was to investigate how immunological dysregulation might be mediating these metabolic disturbances, as well as how exercise might modulate the over-inflammatory state that has been observed in the PCOS population. A review of literature was conducted using PubMed, following the Preferred Reporting Items for Systemic Reviews and Meta-Analysis (PRISMA) guidelines. Results indicated that women with PCOS had higher levels of immunological mediators at baseline when compared to their counterparts without PCOS, suggestive of dysregulated immunological mechanisms. Despite exercise demonstrating benefits in lowering these inflammatory markers in women with PCOS, participants enrolled in exercise programs were unsuccessful in reaching the levels of inflammation comparable to women without PCOS. Further research is warranted to continue investigation into the mechanisms behind the over-inflammatory state observed in women with PCOS and how to best advise patients in managing such inflammation for an improved quality of life.

## Introduction and background

Polycystic ovarian syndrome (PCOS) is a complex and multifactorial disease. It is the leading cause of reproductive and metabolic dysfunction in women and is the most common endocrine pathology in reproductive-aged females worldwide [1, 2]. Despite its large prevalence, impacting up to 15-20% of reproductive-aged females worldwide, and associated risk for serious comorbidities including infertility, insulin resistance, and endometrial cancer, the pathophysiology of PCOS is not well understood, ultimately resulting in difficulties in providing comprehensive treatment guidelines for patients diagnosed with the condition [1, 2].

Recent studies, which will be reviewed in this paper, have found that PCOS has been linked to elevated levels of inflammatory markers, such as C-reactive protein (CRP) and complement factors. This observed over-inflammatory state in patients with PCOS provides new insight into possible mechanisms of pathophysiology, as well as how to potentially adjust treatment guidelines to optimize patient outcomes. With the condition’s significant prevalence, the aim of this review is to further investigate this immunological dysregulation in the PCOS population. Additionally, to investigate how lifestyle modifications, such as participating in regular aerobic exercise, might modulate the disease course and potentially lower insulin resistance and underlying inflammation.

## Review

The goal of this review was to summarize findings from studies examining the role of lifestyle modification in polycystic ovary syndrome (PCOS). The review focuses on the underlying inflammatory changes the body makes in response to PCOS and exercise.

Search strategy

**Figure 1:**
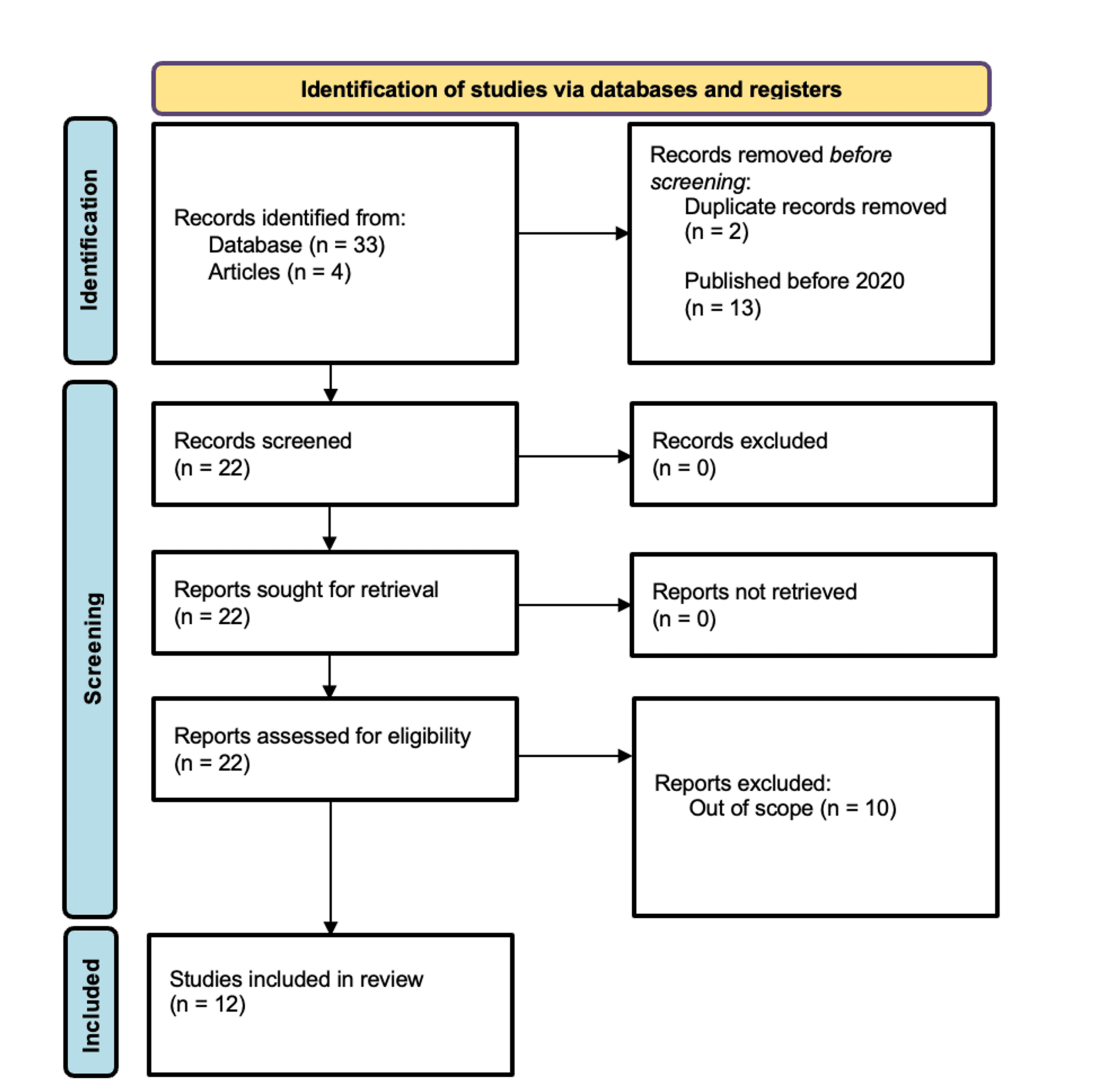
Preferred Reporting Items for Systematic Reviews and Meta-Analyses (PRISMA) diagram of the search strategy to determine studies utilized

One database was used in the search: PubMed. The terms, “exercise AND PCOS AND inflammatory markers”, “exercise AND PCOS AND complement”, “PCOS AND complement dysregulation”, and “PCOS AND inflammatory markers AND complement”, were used, populating a total of 37 articles. Inclusion criteria focused on randomized clinical trials and case-control studies as well as articles from 2020-2024. Exclusion criteria were reviews and papers that were out of this paper's scope. A total of 1,047 patients were therefore observed in the papers that were selected for review. A total of 12 articles were used after analyzing and filtering for exclusion criteria. Figure 1 demonstrates our use of the Preferred Reporting Items for Systemic Reviews and Meta-Analysis (PRISMA).

Pathology and Epidemiology of PCOS

Although PCOS is a well-studied diagnosis, the pathophysiology is still not widely understood. Current literature suggests that the pathophysiology of the disease is largely due to reproductive and metabolic interactions as well as genetic and environmental components [3]. It has also been suggested that PCOS is largely exacerbated by dysfunction of theca cells or of the hypothalamic-pituitary-ovarian axis [3]. These proposed dysfunctions are suggested to be the cause of the hyperandrogenism seen in PCOS patients - a major factor of the disease [3]. Within the most current literature, there were no significant correlations between any particular race and PCOS prevalence. This may be confounded by the underrepresentation of certain races in existing studies and warrants additional studies [4].

The Rotterdam criteria is most commonly used for diagnosing adults with PCOS. It is based on the presence of at least two of the three following criteria: chronic anovulation, hyperandrogenism, and polycystic ovaries. As outlined by Rasquin et al., chronic anovulation is defined as a cycle length of longer than 35 days [1]. Hyperandrogenism can be assessed based on a clinical phenotype of hirsutism or biochemical markers, including free testosterone. Polycystic ovarian morphology is best diagnosed by the use of a transvaginal ultrasound. On ultrasound, the morphology is confirmed if there is the presence of at least 25 follicles measuring between 2-9mm in size within the ovary [1].

Additionally, much like its etiology, the standard of treatment for PCOS is ill-defined and subjective. The current standard of treatment largely involves the use of oral contraceptives in combination with drugs aimed at improving insulin regulation. The use of oral contraceptives is mostly to better regulate the menstrual cycle. Additionally, newer contraceptives have been found to provide additional benefits, such as decreasing hirsutism and acne-phenotypic presentations that are very common among patients with PCOS. Lifestyle modifications have become increasingly important in the management of PCOS patients since higher levels of adipose tissue increase stored androgens. The overall basis for weight reduction is to lower adipose tissue, thus lowering circulating and stored androgens [1].

The primary biological mediators of PCOS are immunologic (complement proteins, complement regulators, CRP), hormonal (androgens like testosterone), and metabolic (insulin, adiponectin). Functions of these mediators are outlined in Table 1.

**Table 1:** Definitions of the primary biological mediators of polycystic ovarian syndrome 17 OHP=17 alpha-hydroxyprogesterone, ATP=adenosine triphosphate, CRP=C-reactive protein, DHEAS=dehydroepiandrosterone sulfate, HOMA-IR=homeostatic assessment model of insulin,  resistance, LH=luteinizing hormone, PCOS=polycystic ovarian syndrome, tT=total testosterone.

Mediator	Physiological Function
Immunologic	
Complement proteins C3, C3b, iC3b, C4, C4a, C4b	The complement cascade is used in a variety of physiological processes that contribute to tissue homeostasis, tissue repair, and immune responses. Functions include identifying and removing pathogens and infected host cells with C3b and C4b, vasodilatory effects with C4a considered an inflammatory mediator, and destroying cell membranes of pathogens with C5b, C6, C7, and C9 components of the membrane attack complex [5].
Complement regulators factor D, factor B, factor H, factor I	These factors prevent other complement proteins from being overactive [5].
CRP	A protein synthesized by the liver in response to inflammation or infection. It is used as a non-specific biomarker for acute or underlying chronic inflammation [6].
Hormonal	
Androgens testosterone, DHEAS, 17-OHP	A precursor to estrogen: conversion from androgens to estrogen occurs primarily in adipose tissue. Androgens promote the progression of primordial follicles, ovarian follicles in the most immature stage, into the growth pool to potentially develop into an ovulated oocyte. It also initiates premature luteinization, impairs the dominant follicle selection, leading to the polycystic ovary phenotype in some PCOS patients [1]. tT includes levels of bound and unbound testosterone [1].
LH	Stimulates ovarian androgen production [1].
Metabolic	
Insulin	The presence of insulin in the blood directs cells to increase the glucose transporters on its cell membrane. This decreases blood glucose levels and increases intracellular glucose concentrations. The cell uses glucose to create ATP, its energy source, or stores it as lipids, glycogen, or proteins. The fate of glucose depends on the cell type and the needs of the cell [1]. High insulin levels can delay the desensitization of LH receptors, enabling LH to act for a longer period of time. This increased duration of action can contribute to menstrual cycle irregularity [1]. Insulin resistance refers to the inability of cells to respond to insulin. Therefore, glucose stays in the blood and cannot be used by the cells. It is most associated with type II diabetes mellitus but also with PCOS [7]. The HOMA-IR is a method of estimating insulin resistance by comparison between patient fasting values and computed predictive values [8].
Adiponectin	Adiponectin is secreted from adipose tissue as a homeostatic regulator of insulin, blood glucose, and the conversion of macrophages from a pro-inflammatory (M1) state to an anti-inflammatory (M2) state. Low levels of adiponectin are associated with insulin resistance [9].

Insulin Resistance and Inflammation

Women with PCOS have an increased metabolic risk with a high association with insulin resistance and chronic inflammation [10]. In a case-control study measuring inflammatory proteins in women with PCOS versus healthy controls, patients with insulin resistance were found to have elevated fasting levels of C3, C3a, and C5/5a, while PCOS patients who were insulin sensitive did not have elevated levels [10]. Comparing controls and PCOS patients, C3 and C4 levels increased similarly after an oral fat tolerance test, but factor H was found to be elevated in women with PCOS, supporting the theory of complement dysregulation in women with PCOS [10].

A similar study from Butler et al. also found an association between alternative complement dysregulation and PCOS patients. Butler utilized two cohorts: obese, insulin-resistant PCOS patients versus non-obese, non-insulin-resistant PCOS patients. The conclusion was that the upregulation of the alternative complement pathway was seen in both cohorts. The dysregulation was exacerbated exclusively in obese PCOS patients [11]. However, upregulation of complement inhibitors was observed in both PCOS groups, which may suggest that physiological processes activate the complement pathways in PCOS [11].

In another study, Dehdashtihaghighat et al. did not find the same association of significantly elevated C3 levels in PCOS patients. Their study included 42 Iranian women with PCOS compared to 42 Iranian women without PCOS. They noted their small sample size as a possible limitation. In this case-control study, they did find that CRP was higher in patients with PCOS compared to controls [12]. A larger case-control study performed by Bannigida et al. had similar results, reporting elevated CRP in women with PCOS regardless of body mass index (BMI), but also noted that insulin resistance was only observed in PCOS patients who were obese [13].

Positive correlations were found between CRP BMI and insulin levels in a research study conducted by Rudnicka et al. White Blood Cells (WBC), CRP, luteinizing hormone (LH), total testosterone (tT), androstenedione, dehydroepiandrosterone sulfate (DHEAS), and 17-OHP levels were significantly increased in the PCOS group while their follicle-stimulating hormone (FSH), estradiol (E2), sex hormone binding globulin (SHBG), and progesterone (PG) were significantly lower compared to healthy controls [14]. The PCOS group also had significantly higher low-density lipoprotein (LDL), glucose, and insulin levels following the oral glucose tolerance test (OGTT) and significantly lower concentrations of high-density lipoprotein (HDL) [14]. Their data is suggestive of a chronic inflammatory state in women with PCOS, with BMI and insulin resistance being the main predicting factors of the increased WBC and CRP levels [14].

Effects of Moderate-Intensity Aerobic Exercise

In a prospective study, researchers investigated the effects of a 20-week aerobic exercise program on the BMI, homeostatic model assessment of insulin resistance (HOMA-IR), and high sensitivity-CRP (hs-CRP) levels of women with PCOS. Their control group consisted of women who were diagnosed with PCOS but did not participate in the home exercise program. They found that the women who were a part of the aerobic exercise training had significantly reduced BMI, HOMA-IR, and hs-CRP levels compared to the control group [15].

Elbandrawy et al. conducted a study to examine the effects of metformin (M group) on inflammatory markers in PCOS women compared to metformin in combination with aerobic exercise (AEM group). The results of the study showed a statistically significant decrease in interleukin-6 (IL-6), tumor necrosis factor-alpha (TNF-alpha), and CRP levels within both the AEM and M groups. When the groups were analyzed in comparison, these decreases were statistically significant from one another. They found a greater decrease in IL-6, TNF-alpha, and CRP levels seen within the AEM group over the M group. Researchers concluded that aerobic exercise was beneficial in reducing IL-6, TNF-alpha, and CRP levels in women with PCOS and should be used as a modality to control the disease [16].

In a study aimed at assessing the effects of aerobic exercise on specific complement factors in women with and without PCOS, women with PCOS were observed to have higher baseline levels of C4b and C3b/iC3b. After completion of an 8-week long exercise program, researchers found a statistically significant decrease in C1q, C3, C4, factor B, factor H, and properdin between the groups. However, the significant reduction of complement-related proteins following moderate aerobic exercise was predominantly found to affect the control group and not the PCOS group. The researchers concluded that the data may suggest dysregulation of the complement system in women with PCOS since the complement factors remained elevated following the completion of the exercise program [17].

A meta-analysis by Moori et al. investigated the effects of exercise on CRP and adiponectin levels. Based on their investigations, they were able to extrapolate that CRP levels had a statistically significant decrease after an exercise program was implemented. However, they found no significant difference in adiponectin levels following exercise training [9].

Gialluria et al. performed a prospective study that investigated the effects of exercise training on inflammatory markers, CRP, and WBCs in women with PCOS. They also measured other cardiovascular measurements, including maximal oxygen consumption (VO2 max), heart rate recovery, and additional metabolic parameters. Following the three-month exercise program, women who had received exercise training had a statistically significant decrease in CRP and WBC levels. Researchers concluded that exercise training was beneficial in improving levels of inflammatory markers in women with PCOS [18].

Discussion

Overall, women with PCOS were found to have a high inflammatory state when compared to women without PCOS. This over-inflammatory state was found to have a positive correlation with insulin resistance. The studies were also able to demonstrate that women with PCOS who participated in moderate-intensity aerobic exercise programs experienced decreased levels of inflammatory markers, such as CRP, WBCs, and complement factors, when compared to women with PCOS who did not participate in the exercise programs, or those who strictly utilized pharmacological therapy. However, the decreases in inflammatory markers seen in women with PCOS who participated in exercise programs did not reach the same baseline level as their counterparts without PCOS.

A major strength of this review was the replicability of the studies. Additionally, each study chose biomarkers that were easily reportable and reproducible. These strengths provide potential benefits for future studies to continue further investigation into the mechanism of the underlying pathophysiology. Moreover, these biomarkers could potentially be used in the future to follow patients’ disease course. Two of the biggest limitations this review found were that the research in this field of study is limited, and sample sizes for most of the studies performed were small. The limited amount of research in the area and small sample sizes present challenges to confidently drawing conclusions about what physicians should be specifically advising patients.

Further research should be conducted to investigate different exercise modalities. Studies could be considered, such as comparing whether high-intensity versus low-intensity aerobic exercise can reduce inflammatory markers more significantly than anaerobic exercises. Additionally, comparing different exercise modalities and the impact on inflammatory markers in women with PCOS should be further investigated, such as resistance training and yoga, against or in combination with aerobic exercise.

## Conclusions

PCOS is a multifactorial disease that can cause excessive inflammation and immune dysregulation. This review found that patients with PCOS who implemented general lifestyle modifications of exercise training had improvements in their insulin resistance and inflammatory markers such as CRP. While encouraging the addition of regular aerobic exercise training to patients with PCOS is advantageous, the patient population could largely benefit from a more specific exercise prescription. However, implementing a specific exercise prescription for PCOS patients still requires further investigation into the effects of different exercise modalities, as well as modifications to the duration and intensity of the modalities. Beyond this review, additional research should gain a more comprehensive understanding of what factors are causing the excess of inflammation in PCOS patients and how to best manage it. Our hope is that future research will lead to decreased fertility complications and increased quality of life for PCOS patients.
